# The effect of music interventions compared to standard-of-care on the prevention of delirium in neurosurgical patients: an analysis of costs and cost-effectiveness based on the MUSYC-trial

**DOI:** 10.1007/s00701-025-06448-0

**Published:** 2025-02-14

**Authors:** Thomas L.A. Dirven, Pablo R. Kappen, Frederike Ten Harmsen van der Beek, Bronno van der Holt, Hans Jeekel, Clemens M.F. Dirven, Arnaud J.P.E. Vincent, Markus Klimek, Marten J. Poley

**Affiliations:** 1 Department of Neuroscience, Erasmus Medical Centre, Room EE1202, dr. Molewaterplein 40, P.O. Box 2040, Rotterdam, South Holland 3000CA The Netherlands; 2 Department of Neurosurgery, Erasmus Medical Centre, Rotterdam, South Holland The Netherlands; 3 Institute for Medical Technology Assessment (iMTA) and Erasmus School of Health Policy & Management, Erasmus University Rotterdam, Rotterdam, South Holland The Netherlands; 4 Department of Haematology, Erasmus MC Cancer Institute, Rotterdam, South Holland The Netherlands; 5 Department of Anaesthesiology, Erasmus Medical Centre, Rotterdam, South Holland The Netherlands; 6 Department of Pediatric Surgery, Sophia Children’s Hospital, Erasmus Medical Centre, Rotterdam, South Holland The Netherlands

**Keywords:** Neurosurgery, Music, Delirium, Cost, Efficiency

## Abstract

**Background:**

Postoperative delirium is a frequent complication with negative consequences for neurosurgical patients. Recorded music has been shown to reduce the incidence of delirium, however its economic benefit remains unclear. This study aimed to investigate the cost-effectiveness of perioperative music in preventing postoperative delirium.

**Methods:**

This study used data from a randomized controlled trial (Clinical Trials.gov; NCT04649450) that compared the effect of perioperative music with standard of clinical care on the occurrence of postoperative delirium in patients undergoing craniotomy at the Erasmus Medical Centre. The primary outcome of this study is the cost-effectiveness of the music intervention. A trial-based cost-effectiveness analysis (CEA) was conducted from a societal perspective. Mean costs were calculated using bootstrapping with 95% confidence intervals. Secondary outcomes included postoperative complications, mortality, cognitive functioning, and quality of life. Costs and patient outcomes were assessed separately for the initial hospital admission and long-term follow-up until 6 months after discharge.

**Results:**

This study included 91 patients in the intervention group and 93 in the control group. On average, medical costs during initial admission were lower, albeit not statistically significant, in the music group compared to the control group (€ 11,819 vs. € 13,106), mostly due to a shorter length of stay. Total costs over the 6-month period were nearly identical between the groups, at € 18,587 and € 18,571 in the music and control group, respectively.

**Conclusions:**

Pre-recorded perioperative music may be a cost-effective intervention for reducing postoperative delirium in neurosurgical patients, possibly by decreasing healthcare utilization and costs during primary admission. Further studies are needed to confirm its potential as a cost-effective intervention.

**Supplementary information:**

The online version contains supplementary material available at 10.1007/s00701-025-06448-0.

## Introduction

Delirium is a medical complication characterized by an acute decline in mental status, affecting attention, awareness, language, cognition, and visuospatial ability [1]. In the neurosurgical population, delirium occurs in 4 to 44% of patients, depending on several factors, such as age, diagnosis and the type of surgery [13, 26, 37, 38]. Delirium is a serious complication as it is in the short-term associated with decreased functional outcome [7], longer hospitalization and higher death rates [8]. In the long term, patients who experience delirium have a higher chance of subjective memory decline and cognitive decline [18]. Furthermore, elderly patients with delirium have a three times higher risk of developing dementia, compared to patients who do not have a delirium [12]. These outcomes are correlated with higher costs. For example, Leslie et al., who analysed the one-year health care costs associated with delirium, showed that patients with delirium incurred an additional cost ranging from $60,516 to $64,421 [24]. Delirium, therefore, impacts both on a clinical and societal level, which justifies intervention studies aimed at lowering the incidence of delirium.

Pharmacologic prevention of delirium is often ineffective, while patients are exposed to potentially serious side effects [35, 25, 36]. Non-pharmacological multi-component approaches, such as the Hospital Elder Life Program (HELP) or the Perioperative Optimization of Senior Health program (POSH), are promising [17, 33]. However, the success of these multi-component strategies depends on the adherence, while implementation is challenging and not always adjusted to the feasibility of nurses or the patients’ needs.

Music is a promising non-pharmacological intervention that has recently gained more attention. Perioperative music interventions have a proven effect on the reduction of risk factors for delirium, such as perioperative pain and anxiety [23, 16, 5, 31] and the requirement of opiates [14]. A randomized controlled trial conducted by our research group, the MUSYC-trial, investigated the effect of music on postoperative delirium in neurosurgical patients [22]. Our initial analysis of the trial, focusing on the time horizon of hospital admission, showed a significant reduction on delirium occurrence measured by DOSS (Delirium Observational Screening Scale), while a non-significant trend was found using the DSM-V diagnostic criteria [22]. Hence, perioperative music proved a promising intervention for reducing postoperative delirium in neurosurgical patients. However, the relevance of these findings remains unclear as the long-term clinical results and its healthcare related costs have not been evaluated. Therefore, the evidence on the cost-effectiveness of music interventions in neurosurgery is unknown.

Over the last decades, economic evaluations of healthcare have evolved into an important and active area of research. Amid the current healthcare landscape of rising costs and pressure on budgets, it is increasingly important that new interventions offer good value for money [32, 11]. However, economic evaluations have hardly been applied in the field of music interventions in healthcare. Therefore, in this study we investigate the costs and benefits of these interventions with an evidence-based justification, which supports insights for the implementation.

The aim of this study was to analyse the cost-effectiveness and the long-term clinical effects of a music intervention on the prevention of delirium in a neurosurgical population.

## Methods and materials

### Randomized controlled trial

The MUSYC-trial is a single centre, prospective randomized controlled trial conducted at the department of neurosurgery in Erasmus University Medical Centre Rotterdam, the Netherlands. The trial compared the effects of music administered before, during and after craniotomy with standard clinical care. The music intervention was carried out 30 min preoperatively via over-ear headphones, during the operation via in-ear earphones and postoperatively 30 min twice a day until postoperative day 3 via over-ear headphones. For participants in the music group, music was available via a tablet with access to a platform with preselected music playlists and additional tracks. The adherence of the intervention was noted by nurses in a diary of each subject. Eligible patients were ≥ 18 years-old, undergoing craniotomy, and proficient in the Dutch language. Further details of the protocol and the performed trial have been published previously [19, 22] and are reported in several registries.

### Economic analysis

Following well-established methodologies for economic evaluation in healthcare [34, 11], an economic analysis was done using the technique of trial-based cost-effectiveness analysis (CEA). This type of analysis provides information on the cost-effectiveness of interventions in terms of additional costs to achieve a specific health outcome. The economic evaluation was performed from a societal perspective, which considers all relevant costs, no matter on whom they fall. For instance, not only were costs during the primary admission assessed, but also costs regarding decreased productivity of the patient after admission. The time horizon of the CEA was the 6 months follow-up period of the MUSYC trial. Given this relatively short follow-up period, there was no need to discount costs and effects arising in the future. In accordance with the study design of the MUSYC-trial, the CEA focused on the modified intention-to-treat population (mITT), which implies that the analysis did not include patients who were excluded after randomization due to not meeting the in- and exclusion criteria (such as withdrawal of consent, cancelled operations, or undergoing a procedure other than craniotomy). The economic evaluation was reported according to the Consolidated Health Economic Evaluation Reporting Standards (CHEERS), as shown in Supplementary Table S1.

### Data extraction

Baseline characteristics of the study participants were recorded and data on healthcare utilization and health outcomes were extracted in detail of each individual participant, using the electronic health records and validated questionnaires. Data were collected during admission and during both follow-up moments, i.e., 3 months and 6 months after admission.

### Costs

Consistent with the societal perspective of the analysis, four types of costs were considered: direct medical, indirect medical, direct non-medical, and indirect non-medical costs.

*Direct medical costs* referred to the costs of the resources used during the primary hospital admission, such as hospital admission days, surgeries, and medications. Additionally, for the intervention group, we added the costs of the music intervention, covering all products used for the music intervention (including headphones, earbuds, and tablets). *Indirect medical costs* encompassed the costs of healthcare use after hospital discharge (until 6 months), including care provided by Erasmus MC (e.g., readmissions and reoperations) and by other healthcare providers (e.g., general practitioners and homecare). These costs were measured using the iMTA Medical Consumption Questionnaire (iMCQ) [3]. For both direct and indirect medical costs, the analysis included costs related to the (indication for) neurosurgery, to treating delirium, or to adverse consequences due to delirium. Data on healthcare utilization were combined with unit costs to generate patient-level costs. Unit prices were mainly based on reference prices taken from the Dutch Manual for Costing Research [15] and on other sources (see Supplementary Table S2). The calculation of music intervention costs is presented in Supplementary table S3.

*Direct non-medical costs* referred to the costs of time spent by informal caregivers, such as partners and family members, in providing household assistance, personal care, or practical help to the patient. These costs were valued using the proxy good method, which implies that they were based on the replacement costs of household care (i.e., costs of € 15.64 per hour).

*Indirect non-medical costs* encompassed productivity losses regarding paid and unpaid work of the patient. Productivity losses regarding paid work included hours lost due to absence of work (absenteeism) and reduced productivity while being at work (presenteeism). Regarding unpaid work, foregone activities such as household tasks or volunteer work were considered. Indirect non-medical costs were measured using the iMTA Productivity Cost Questionnaire (iPCQ) [4]. These costs were valued according to the friction cost method, using productivity costs of € 38.97 per hour of paid work. Regarding unpaid work, the proxy good method was applied, which implies that these costs were based on the replacement costs of household care (i.e., costs of € 15.64 per hour).

All costs were calculated in Euros (€) and expressed in 2021 prices.

### Secondary outcomes

The secondary outcomes were the long-term implications of the delirium, including complications, mortality, cognitive functioning, and health-related quality of life. The type of complication, e.g. wound infection, increased intracranial pressure or epilepsy, was assessed during admission. Mortality was measured during the follow-up at 6 months. Cognitive function was assessed with the Montreal Cognitive Assessment tool (MOCA) [30] at baseline, 3 and 6 months. It is a screening instrument for mild cognitive impairments. Nine different domains are tested: executive functioning, language, attention, concentration, memory, visuoconstructional skills, conceptual thinking, calculations, and orientation. The highest attainable score is 30. A score above 26 is considered as normal cognitive functioning. Health-related quality of life was assessed using the EuroQol EQ-5D questionnaire [6]. This questionnaire is a preference-based, widely used instrument for measuring the quality of life, based on five health dimensions, e.g. mobility, self-care, usual activities, pain/discomfort and anxiety/depression.

### Cost-effectiveness

For the diagnosis of delirium, the Delirium Observational Scoring Scale (DOSS) and the DSM-V were used. Firstly, the DOSS, a validated 13-item tool with a total score ranging from 0 to 13, was performed three times per day by nurses as part of standard care. A mean DOSS score of 3 or higher indicated a higher probability of delirium. CT imaging was not routinely included in the protocol for subjects with an elevated DOSS. However, in clinical practice, CT imaging is frequently performed in the event of neurological deterioration, which may lead to an elevated DOSS, in order to rule out complications. Secondly, for patients with a daily mean DOSS of ≥ 3, without a detected neurosurgical complication, a psychiatrist was consulted to confirm the clinical diagnosis of delirium according to DSM-V criteria. For the CEA, we use the outcomes of DSM-V of the MUSYC trial, see the previous publication [20].

The final outcome measure for the CEA was the incremental cost-effectiveness ratio (ICER), which was calculated by dividing the difference in costs between the groups by the difference in effects on delirium diagnosis, unless one treatment dominated the other (i.e., has similar or lower costs and greater effects). The ICER was labelled as incremental costs per case of delirium diagnosis prevented.

### Statistical analysis

The sample size was based on the primary trial [22] and protocol [19], which focused on delirium prevention with music: we expected a delirium incidence of 24.2–32.4% using the DOSS [21] and considered a 60% reduction clinically relevant. Using a power of 80%, a two-sided significance level (α) of 0.05, and assuming a loss to follow-up of 5%, we estimated a target sample size of 189 participants. Categorical data were compared between both groups using the Chi-square test or Fisher’s exact test whichever applicable. Continuous data were checked for normality, using the Shapiro-Wilk test. When the data was not normally distributed, the Mann-Whitney-U test was used, and the independent T-test was used for parametric data. Due to nonparametric values, mean costs were calculated using bootstrapping, with 95% confidence intervals reported. For all different healthcare and costs variables, multiple statistical tests had to be executed. Therefore, a Bonferroni correction was performed. The significance level was adjusted for the amount of tests and reported when applicable. Furthermore, to express the uncertainty around the point estimates for the ICER, bootstrapped ICERs were calculated using 5,000 samples. The bootstrap replicates were plotted on cost-effectiveness planes, which were created using ‘package BCEA’ in Rstudio Team (2020). Data were analysed using IBM SPSS Statistics for Windows, Version 28.0.

## Results

**Fig. 1:**
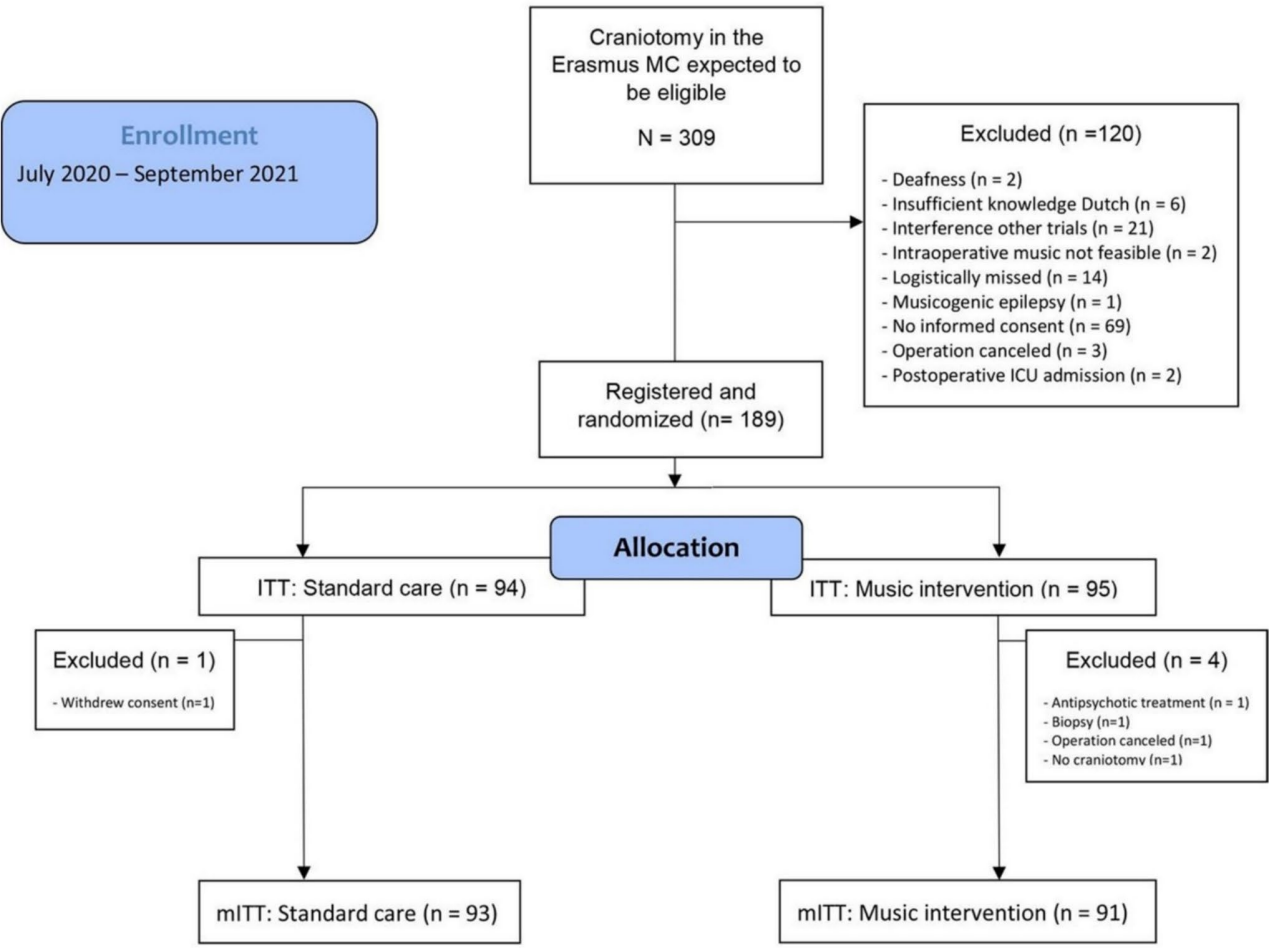
CONSORT Flowchart. Cited from: Kappen PR, Mos MI, Jeekel J, Dirven CMF, Kushner SA, Osse RJ, Coesmans M, Poley MJ, van Schie MS, van der Holt B, Klimek M, Vincent AJPE. Music to prevent deliriUm during neuroSurgerY (MUSYC): a single-centre, prospective randomised controlled trial. BMJ Open. 2023 Jun 27;13(6):e069957. doi:10.1136/bmjopen-2022–069957. PMID: 37,369,412; PMCID: PMC10410844

In the primary MUSYC-trial, a total of 189 patients were randomized. After excluding patients who did not meet the in- and exclusion criteria, 91 patients in the music group and 93 patients in the control group remained. For a flow diagram of patient inclusion, see the previous published trial [22] and Fig. 1. Baseline characteristics (Table 1) revealed that the music group and control group were generally comparable, with a median age of 60 (IQR 49–69). The proportions of female patients (41 vs. 50%), patients with a psychiatric medical history (6 vs. 15%), and those with reported alcohol or drugs abuse (4 vs. 10%) were lower in the music group compared to the control group. Delirium prior to admission (1 vs. 3%) was comparable in both groups. The most frequent type of indication for surgery was oncology (86%) and median baseline cognition (MoCA) was 24 (IQR 20–27). In total, 32 patients had an increased DOSS [20]. In three of these patients with an increased DOSS score, this was explained by a neurosurgical complication, confirmed on radiology: two patients with infarction after a vascular procedure with hemiparesis and decreased attention, while the third patient had a subdural haematoma that required evacuation in the operating room.

**Table 1:** Baseline characteristics

	*N*	Control (*n* = 93)	Music (*n* = 91)
Age (years)*	184	61 (50–69)	59 (48–69)
Female	184	46 (49.5)	37 (40.7)
BMI (kg/m^2^) *	184	26.2 (24.4–29.7)	25.8 (23.4–28.3)
Medical history:			
Somatic †	184	78 (84)	71 (78)
Psychiatric	184	14 (15)	5 (6)
Delirium prior to admission	184	3 (3)	1 (1)
Dementia	184	0	0
Medication **	184	15 (16)	14 (15)
Intoxication:			
Alcohol or drugs §	184	9 (10)	4 (4)
Prehospital functional status* ¶			
KPS	184	70 (10–80)	70 (50–90)
MRS	184	1 (0–2)	1 (1–2)
Quality of life * ‡	134	70 (60–80)	65 (50–78.75)
Cognitive function (0–30) * ††	134	25 (21–27)	24 (19.25–26.75)
Disease type:			
Oncological	184	80 (86)	78 (86)
Vascular	184	12 (13)	12 (13)
Other	184	1 (1)	1 (1)
Music importance* ‡‡	174	8 (7–8)	7 (6–8)
Played an instrument	162	18 (22)	20 (25)

Values are in number (%). *Values are reported in median (IQR) † Somatic history, including: systematic disease (e.g. hypertension or diabetes mellitus) currently treated by medication or previous surgery with general anaesthesia **Assessed as medication known to induce delirium prior to admission, including morphine, sleep medication, atropine and antidepressants. § Reported abusive use of alcohol and drugs ¶ Functional performance of patients, using Karnofsky Performance Scale (ranging from 100/’no complaints’ to 0/’death’) and Modified Ranking Scale (ranging from 0/’no symptoms to 5/’death’) ‡ Assessed using EuroQol-5-D †† Cognitive function measured by the Montreal Cognitive Assessment ‡‡ Assessed using an ordinal scale, ranging from 0–10 (0 is ‘not important’ to 10 is ‘most important’)

### Resource use and costs

Resource use and its associated costs, across all four cost categories, are shown in Tables 2 and 3, with further details in Supplementary Table S4. With respect to *direct medical* costs (i.e. *during primary hospital admission)*, no statistically significant differences in healthcare use or costs were observed between the music and control group. However, some relevant differences were observed: the operation duration (308 vs. 329 min), length of stay (6.4 vs. 7.4 days), number of consultations (1.1 vs. 1.7), and the use of certain medications (especially naproxen and benzodiazepines) were all lower on average in the music group. This resulted in € 1,287 lower costs of the primary admission in the music group, compared to the control group (€11,819; CI 95% 9,945 − 14,582 vs. €13,106; CI 95% 11,101 − 15,915), mainly caused by fewer hospital days (€ 720).

**Table 2:** Resource use

	Control			Music			
	N*	Total number (Mean)	Median (IQR)	N*	Total number (Mean)	Median (IQR)	P-value
**Direct medical resources (during primary admission)**							
Duration of operation (minutes)	93	30,571 (328.72)	283 (226.50–405)	91	28,046 (308.20)	281 (205–358)	0.30
Length of stay (days) †	93	691 (7.43)	4 (3–7)	91	583 (6.41)	4 (4–7)	0.10
Radiology **	93	218 (2.34)	2 (2–2)	91	194 (2.13)	2 (1–2)	0.14
Laboratory test	93	898 (9.66)	7 (5–10)	91	944 (10.37)	7 (5–9)	0.59
Medical consultations ¶	93	154 (1.66)	0 (0–1)	91	97 (1.07)	0 (0–0)	0.55
Paramedical consultations ¶	93	242 (2.6)	1 (0–2)	91	179 (1.97)	0 (0–0)	0.62
Medication dose (milligram): ‡							
Naproxen	93	10,250 (110.22)	0 (0–0)	91	5750 (63.19)	0 (0–0)	0.06
Oxynorm	93	485 (5.22)	0 (0–5)	91	315 (3.46)	0 (0–5)	0.98
Oxycontin	93	280 (3.01)	0 (0–0)	91	350 (3.85)	0 (0–0)	0.69
Haloperidol	93	149.5 (1.61)	0 (0–0)	91	34 (0.37)	0 (0–0)	0.52
Benzodiazepines	93	478 (5.14)	0 (0–0)	91	111 (1.22)	0 (0–0)	0.08
**Indirect medical resources (follow-up at 6 months)**							
Extracted from Electronic Health Record:							
Readmissions ††	93	21 (0.23)	0 (0–0)	91	34 (0.37)	0 (0–0)	0.83
Reoperations ††	93	14 (0.15)	0 (0–0)	91	16 (0.18)	0 (0–0)	0.77
Radiology **	93	126 (1.35)	1 (1–2)	91	138 (1.52)	1 (1–2)	0.46
Extracted from questionnaires:							
Outpatient consultations ‡‡	38	285 (7.5)	5 (2–8.5)	37	327 (8.84)	4 (1.5–8.0)	0.65
Homecare (hours) ¶¶	38	361 (9.5)	0 (0–0)	37	430 (11.62)	0 (0–0)	0.48
Visit to institution ^1^	38	53 (1.39)	0 (0–0)	37	147 (3.97)	0 (0–0)	0.15
Overnight stay in an institution ^2^	38	91 (2.39)	0 (0–0)	37	2 (0.05)	0 (0–0)	0.30
**Direct non-medical resources (follow-up at 6 months)**							
Time of informal caregivers (hours) ^3^	38	3563 (93.76)	0 (0–73.5)	37	6630 (179.19)	30 (0–218.50)	0.07
**Indirect non-medical resources (follow-up at 6 months)**							
Productivity losses regarding paid work:							
Number of hours lost (absenteeism) ^4^	38	2221.71 (58.47)	0 (0–108)	37	2017.43 (54.53)	0 (0–62.86)	0.82
Number of hours of reduced productivity while being at work (presenteeism) ^5^	38	200 (5.26)	0 (0–0)	37	379.40 (10.25)	0 (0–0)	0.17
Productivity losses regarding unpaid work: number of hours lost ^6^	38	2383 (62.71)	0 (0–7)	37	1804 (48.76)	0 (0–44)	0.13

All p-values are measured using Mann Whitney-U Test, due to non-parametric data. Bonferroni-corrected alpha is 0.002, i.e. p-values smaller than 0.002 is defined as statistically significant. *N: Number of patients with available data † Length of stay during admission on nursing ward, intensive care unit and post anaesthesia care unit **Number of performed radiologic tests, including MRI, CT and X-rays ¶ Inter-collegial consultations of medical professionals (e.g. psychiatrists, internal medicine doctors or rehabilitation doctors) or paramedical professionals (e.g. dietitian, physiotherapists, ergo therapists) in hospital during admission ‡ Administered medication dose during admission †† In Erasmus Medical Centre, this patients data of other centres is unknown ‡‡ Reported outpatient consultations by patient of general practitioner, occupational physician, social worker, psychologist or psychiatrist ¶¶ Reported homecare by patient, including household aid, personal care, nursing or a combination of these types of care. ^1^ Reported visits by patient, including visits to facility care centre, rehabilitation centre and psychiatric unit ^2^ Reported overnight stays by patient, including stays at facility care centre, rehabilitation centre, psychiatric unit or other stays. ^3^ Reported time of informal caregivers, related to homecare, personal care, practical care or a combination of these types of care ^4^ Productivity loss reported by patient, due to absenteeism of the ill patient ^5^ Productivity loss reported by patient, due to inability of efficient work as a result of health complaints. ^6^ Unperformed unpaid work reported by patient, such as cleaning work at home or volunteer work

**Table 3:** Costs

	Control		Music		Difference (music - control)	*P*-value
	N	Mean Costs in € (95% CI)	N	Mean Costs in € (95% CI)	Mean Costs(€)	
**Direct medical costs (During primary admission)**						
Operation *	93	7049 (6418–7718)	91	6587 (5980–7196)	−462	0.37
Hospital length of stay †	93	5089 (3489–7248)	91	4369 (3001–6529)	−720	0.96
Radiology **	93	423 (366–490)	91	399 (340–482)	−24	0,25
Laboratory tests	93	237 (194–287)	91	257 (175–406)	20	0.60
Consultations	93	180 (93–290)	91	119 (58–198)	−61	0.60
Medication	93	1 (0.39–0.94)	91	0 (0.23–0.72)	−1	0.40
Music intervention §	93	0 (0–0)	91	7 (7–7)	7	< 0.001
*Total direct medical costs*	93	13,106 (11,101 − 15,915)	91	11,819 (9,945 − 14,582)	−1,287	0.18
**Indirect medical costs (Follow-up at 6 months)**						
Extracted from Electronic Health Record:						
Readmissions at ErasmusMC †	93	623 (315–975)	91	1282 (516–2260)	659	0.80
Reoperations at ErasmusMC *	93	303 (122–527)	91	622 (251–1087)	319	0.71
Radiology §	93	280 (237–321)	91	312 (263–360)	32	0.49
Extracted from questionnaires:						
Outpatient consultations ¶	38	396 (226–619)	37	418 (232–684)	22	0.52
Homecare	38	499 (6–1356)	37	447 (53–1061)	−52	0.50
Visit to institution ‡	38	219 (0–653)	37	484 (120–958)	265	0.22
Overnight stay in an institution ††	38	793 (0–2125)	37	28 (0–88)	−765	0.30
*Total indirect medical costs*	38	2656 (1132–4629)	37	2035 (1066–3357)	−621	0.48
**Direct non-medical costs (Follow-up at 6 months)**						
Time of informal caregivers ‡‡	38	1466 (572–2623)	37	2803 (1097–5406)	1337	0.07
**Indirect non-medical costs (Follow-up at 6 months)**						
Paid work:						
Absenteeism ^1^	38	2278 (1036–3639)	37	2125 (882–3602)	−153	0.82
Presenteeism ^2^	38	205 (0–815)	37	400 (13–980)	195	0.17
Unpaid work ^3^	38	981 (206–1975)	37	763 (347–1332)	−218	0.13
*Total indirect non-medical costs*	38	3464 (1667–5655)	37	3287 (1507–5496)	−177	0.49
**Costs during primary admission (Total direct medical costs)**	93	13,106 (11101–15915)	91	11,819 (9945–14582)	−1287	0.18
**Costs during follow-up**	38	7587 (4547–11134)	37	8124 (4687–12356)	537	0.78
**Total costs**	38	18,587 (14740–22482)	37	18,571 (14457–23618)	−16	0.78

Mean costs calculated based on healthcare utilization of patients related to indication of neurosurgery and or related to (consequences of) delirium in control and music group patients. Costs are measured using bootstrapping, reported as means (95% confidence intervals). P-values were calculated using Mann-Whitney U tests, due to non-parametric data. Bonferroni-corrected alpha is 0.002, i.e. p-values smaller than 0.002 is defined as statistically significant. *including costs for duration, surgeons, anaesthesiologists, supporting staff, operation room. We assumed that every operation had 1 neurosurgeon and 1 anaesthesiologist, 1 neurosurgery resident and 1 anaesthesiology resident † Costs are calculated with respect to admission on nursing department, post-anaesthesia care unit and intensive care unit **Costs of performed radiologic tests, including MRI, CT and X-rays § Calculated price for music intervention per patient ¶ Cost calculated by reported outpatient consultations by patient of general practitioner, occupational physician, social worker, psychologist or psychiatrist ‡ Costs based on reported visits to facility care centre, rehabilitation centre and psychiatric unit †† Costs based on reported overnight stays, including stays at facility care centre, rehabilitation centre, psychiatric unit and other stays ‡‡ Costs based on reported time of informal caregivers ^1^ Costs based on productivity loss reported by patient, due to absenteeism of the ill patient ^2^ Costs based on productivity loss reported by patient, due to inability of efficient work as a result of health complaints ^3^ Costs based on unpaid work reported by patient, such as cleaning work at home or volunteer work

With respect to indirect medical costs (covering the period after discharge until 6 months of follow-up), no significant differences were observed in healthcare use or costs between the music and control group. However, in the music group, the numbers of readmissions, reoperations, and radio-diagnostic procedures were slightly higher. Similarly, patients in the music group had slightly more outpatient consultations, received more homecare, and had more visits to institutions after hospital discharge, although they had fewer overnight stays in institutions (i.e., facility care, rehabilitation centres, or psychiatric units). Overall, indirect medical costs appeared somewhat lower in the music group (€ 2,035 vs. € 2,656 in the control group), primarily due to lower costs of stays in institutions.

In the music group, informal caregivers spent more time on providing care and assistance, compared to the control group (averages of 179 vs. 94 h, respectively). As a result, direct non-medical costs were higher in the music group, with a difference of € 1,337, although this difference did not reach statistical significance.

Finally, there were no obvious differences between the groups regarding productivity losses, although the number of lost hours of unpaid work seemed somewhat lower in the music group. Overall, indirect non- medical costs were slightly lower in the music group (€ 3,287 compared to € 3,464 in the control group).

Total costs, calculated by summing all four cost categories, were minimally lower in the music group compared to the control group (€18,571; CI 95% 14,457 − 23,618 vs. €18,587; CI 95% 14,740 − 22,482).

### Patient outcomes

Table 4 shows the patient outcomes in both study groups. The primary comparison of patient outcomes between both intervention groups are illustrated in the prior publication[ref]. No other clinically relevant differences were observed between the study groups. The incidence of complications, of which internal medicine complications and neurologic deterioration were most common, was similar between the music and the control group, as was the mortality rate. Over time, cognitive functioning in both study groups decreased slightly compared to baseline, but no differences were observed between both groups. In both groups, hardly any changes in quality of life over the follow-up period were found.

**Table 4:** Patient outcomes

	Control group (*n* = 93)	Music group (*n* = 91)	*P*-value
	Number (%)	Number (%)	
Mortality §	15 (16)	11 (12)	0.43*
Complications: ¶			
Intra-operative	3 (0.03)	4 (0.04)	0.68
Internal medicine	11 (0.12)	12 (0.13)	0.68
Limb fractures	2 (0.02)	0 (0)	0.16
Neurologic deterioration	16 (0.17)	11 (0.12)	0.42
Epilepsy	2 (0.02)	2 (0.02)	0.98
Wound/Infection	1 (0.01)	6 (0.07)	0.09
Increased ICP	5 (0.05)	3 (0.03)	0.49
Cognitive functioning: †			
Difference baseline to 3 months	−6.5(−8.0;−2.0)	−5.0 (−7.0;−3.0)	0.17
Difference baseline to 6 months	−5.0 (−7.0;−3.0)	−5.0 (−8.0;−1.0)	0.86
Quality of life: ††			
Difference baseline to 3 months	0 (−0.17;0.20)	0 (−0.17;0.19)	0.51‡‡
Difference baseline to 6 months	−0.2 (−0.28;0.19)	0 (−0.38;0.16)	0.62

Values are reported in number (%) and p-values are calculated using Mann-Whitney U test, unless otherwise indicated. *P-values were assessed using Chi-square-test § Measured at 6 months follow-up. ¶ Reported in number (mean). † Cognitive functioning is reported in median (i.q.r.) and data was present of 95 patients on 3-months-follow-up and 89 patients on 6-months-follow-up in total. †† Quality of life is reported in median (i.q.r.) and data was present of 81 patients on 3-months-follow-up and 71 patients on 6-months-follow-up in total. ‡‡Analysed using independent T-Test

### Cost-effectiveness

The cost-effectiveness of the music intervention was assessed in two ways. First, cost-effectiveness was analysed considering the total costs over the entire study period (next to the incidence of delirium), including only the 75 patients with complete follow-up (38 in the control group and 37 in the music group). Second, cost-effectiveness was calculated from the short-term perspective of the initial hospital admission, considering only direct medical costs, and based on all 184 patients.

**Fig. 2:**
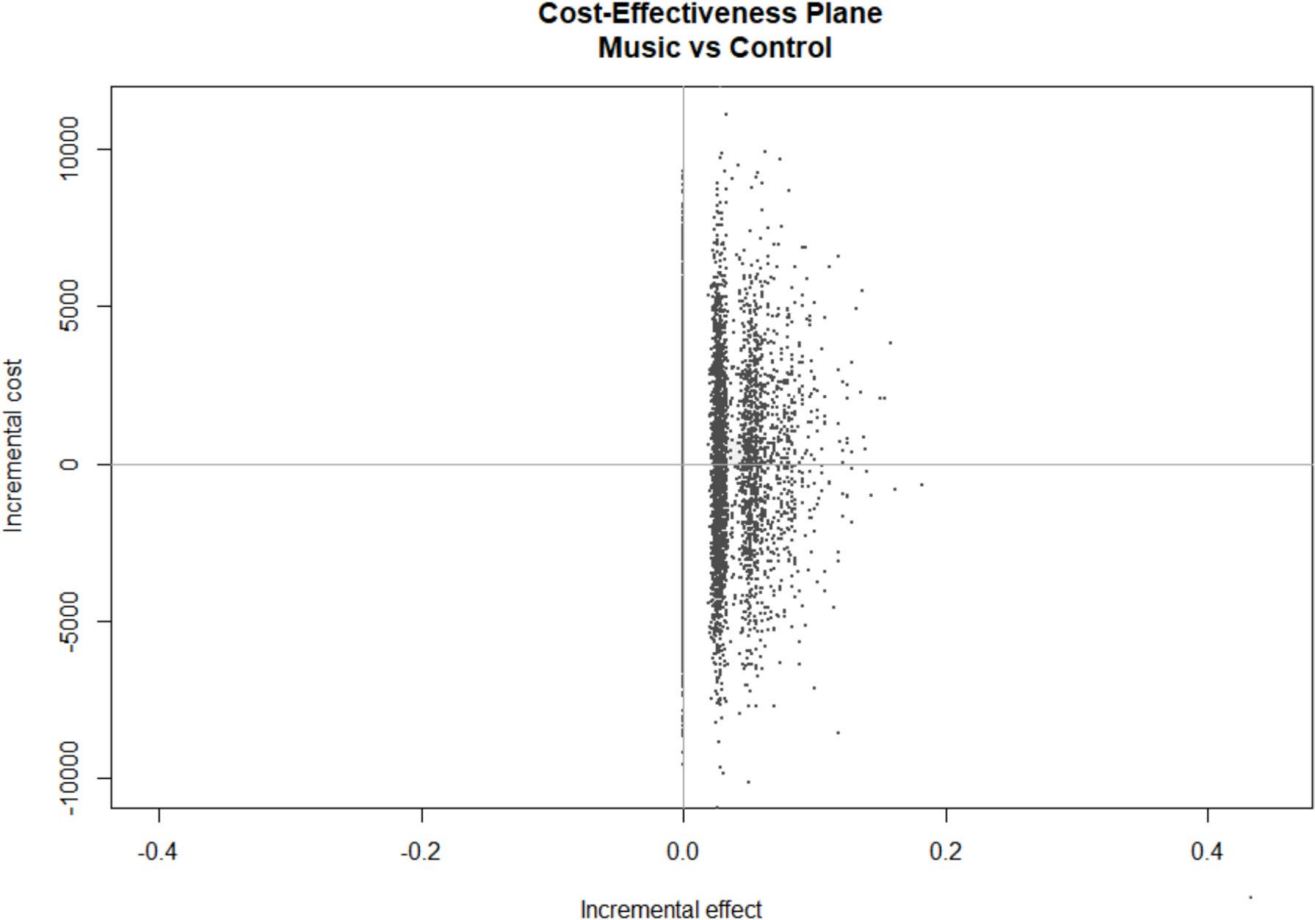
Cost-effectiveness plane of complete study period. Scatter plot showing the bootstrapped replications for the incremental costs and effects, considering the entire study period. X-axis (Incremental Effect): a negative value indicates a reduction in delirium rates due to the intervention. Y-axis (Incremental Cost): a negative value indicates cost savings due to the intervention

For the subgroup of 75 patients with complete follow-up, the total costs in the music intervention group were slightly lower (€ 16) compared to the control group, as previously mentioned. This translates into an ICER of -€ 593, which would suggest that for each additional patient with delirium, € 593 could be saved. There was some uncertainty surrounding the estimates of expected incremental cost (in Euros) and expected incremental effect (occurrence of delirium) associated with the music intervention, as shown in a cost-effectiveness plane (Fig. 2). The figure demonstrates considerable uncertainty regarding the existence of cost savings (ranging from -€10,908 to €11,989), while no points fell in in the western quadrants of the cost-effectiveness plane.

**Fig. 3:**
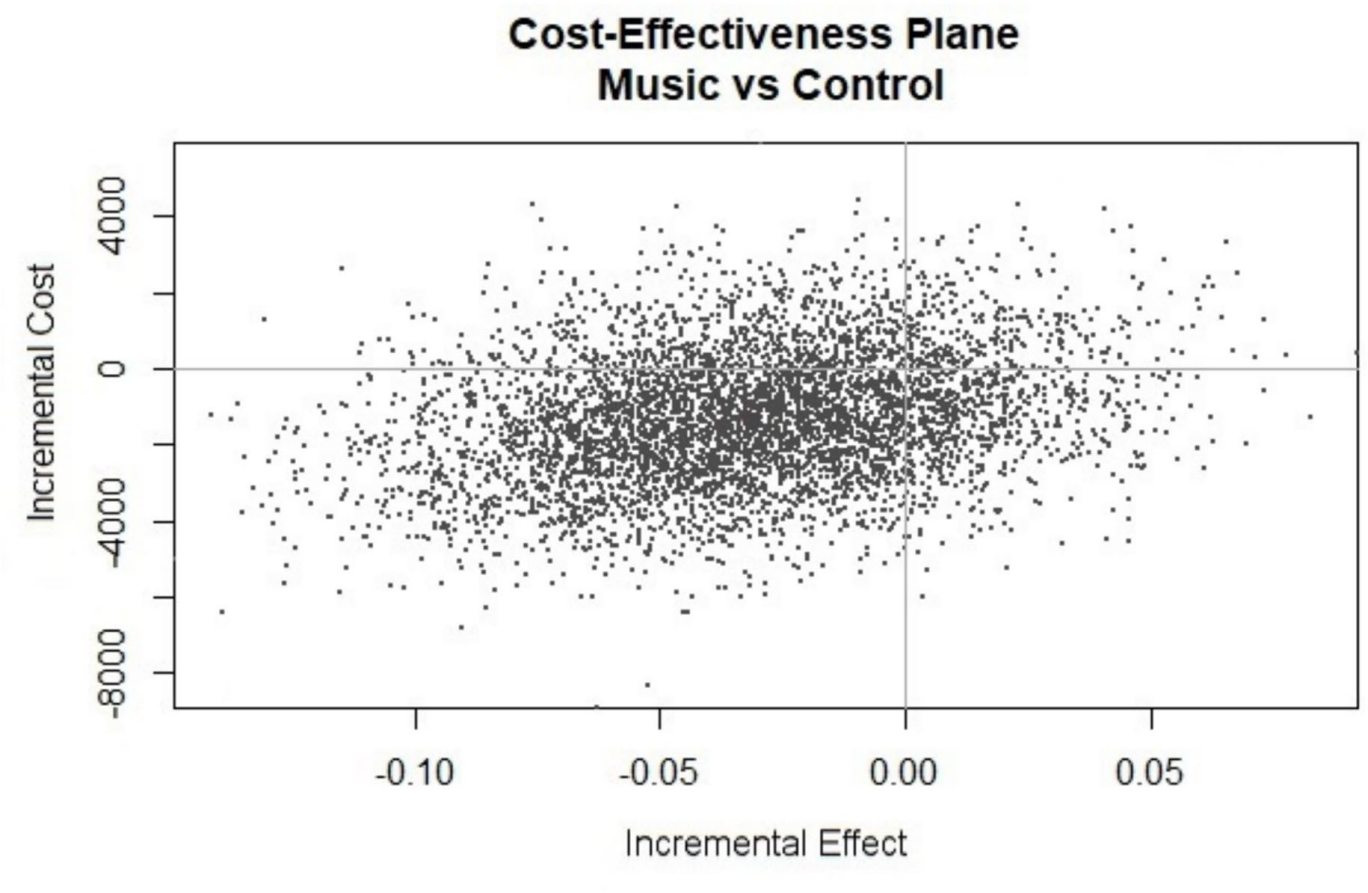
Cost-effectiveness plane primary admission. Scatter plot showing the bootstrapped replications for the incremental costs and effects, considering the period of the primary admission. X-axis (Incremental Effect): a negative value indicates a reduction in delirium rates due to the intervention. Y-axis (Incremental Cost): a negative value indicates cost savings due to the intervention

From the short-term perspective of the initial hospital admission and considering all 184 patients, it can be concluded that the costs in the music group were somewhat lower compared to the control group (€ 11,819 versus € 13,106), while there was a trend towards a lower occurrence of confirmed delirium [20]. So, in health-economic terms, the music intervention was deemed to be the dominant strategy, and therefore no ICER was calculated. Again, there was some uncertainty surrounding the estimates of incremental costs and effects, as is presented in a cost-effectiveness plane (Fig. 3). With regard to effectiveness, the location of the incremental cost-effect pairs indicates that there is little uncertainty regarding the existence of a delirium benefit associated with the intervention. However, the location and spread of the points indicates that there is some uncertainty regarding the existence of cost savings with the music intervention, because not all points were located below the horizontal axis. The magnitude of the cost savings varied from -€8,619 to €5,874. Overall, 66% of the points were located in the southwest quadrant of the cost-effectiveness plane, indicating that the music intervention is less costly and more effective than usual care for the prevention of delirium.

## Discussion

### Main results

This article has focused attention on the cost-effectiveness of a music intervention in patients with neurosurgical conditions, compared to standard care. Although no statistically significant differences were observed, healthcare utilization during the primary admission seemed to be lower in the patients receiving the music intervention, with shorter lengths of stay and fewer consultations, which may have led to lower costs. This economic benefit may have resulted from the clinical benefit of less delirium in the music group. However, this possible benefit by music was not sustained in the long-term. At 6 months follow-up, healthcare utilization after hospital discharge seemed to be slightly higher in the music group, with the exception of overnight stays in institutions, and patients in the music group received somewhat more care and assistance from informal caregivers. Overall, total costs over the 6-month follow up period were nearly identical between the music and the control group. However, it must be kept in mind that this calculation of total costs was more uncertain due to the smaller sample size resulting from loss to follow-up. The observed trends towards a lower incidence of delirium [20] and lower or at least identical costs, suggest that the studied music intervention may offer value for money in neurosurgical patients.

### During admission

The lower costs of the initial hospital admission found in the music group were mainly due to a shorter length of stay. This might have been caused by the reduced incidence of delirium, as other non-neurosurgical studies showed a correlation between delirium and prolonged length of stay [29, 39, 10]. Mccusker et al. suggested possible explanations for the relationship between delirium and prolonged length of stay [28]. Firstly, delirium can lead to less mobilization, impeding discharge. Secondly, delirium may be triggered by an underlying complication, which could be the cause for the longer hospitalization. Lastly, the diagnosis of delirium could require further evaluation or assessments, which may contribute to increased length of stay. This may be particularly relevant in our population, as neurologic symptoms often overlap with delirium criteria and may need further evaluation. The beneficial effect of music on length of stay has not been confirmed in prior studies. For example, a systematic review by Fu and colleagues found no correlation between music interventions and length of stay (LOS) [14]. The discrepancy with our findings may have been caused by the fact that a substantial part of their cohort (44%) underwent minor surgery in outpatient clinics.

Additional costs reductions were achieved by fewer consultations in the music group. This is intuitive, since psychiatric evaluation is mandated for delirium diagnosis in this complex group, as overlap of primary neurologic symptoms challenges the interpretation of nurse-implemented screening tools. Lastly, a shorter operation duration was observed in the music group. It is hard to see a clear relationship with the music intervention, apart from the possibility that sedation induction might have been more eased, and perhaps time-efficient, in the music group [14]. Moreover, operations may have been different between the study groups due to smaller tumour sizes or more superficial locations. However, this is speculative, as we did not study these covariates.

The costs of administering the music intervention were rather low, at € 7 per patient. This can be explained by the fact that we used pre-recorded music and did not consult a paid music therapist for the development of patient-specific music selection and patients could choose their own favourite music. This made our music intervention less expensive compared to previous studies that did use music therapists [9, 2].

### After admission

Healthcare use from discharge until 6 months after surgery was not affected by the music intervention during admission. Although not statistically significant, a relevant finding was that more wound infections were observed in the music group, which may be attributed to the nearby position of the headphones and the cranial site of surgery. Additionally, more healthcare related time of informal caregivers was observed in the music group. This might have been caused by the lower rate of patients discharged towards other institutions for rehabilitation (such as facility care centres, rehabilitation centres or psychiatric units). Hence, despite a shift in care from health care personnel in institutions to home-situated informal caregivers, overall, the healthcare costs for the patients in the music and control group were comparable.

No differences in quality of life or cognitive function were seen at 3 or 6 months after hospital discharge. Previously, McCrary et al. found in their meta-analysis improvements in health-related quality of life associated with music interventions [27]. However, the timing of health-related quality of life assessment in relation to the last intervention was not reported in this study. In our trial, there was a relatively long period between the last music intervention and the assessments of quality of life and cognitive function, i.e. 3 and 6 months after surgery. The duration of the effect of a music intervention is unclear and it is possible that the effect on these outcomes was diminished over time and was not measurable during our assessments.

### Limitations

This is the first study assessing the cost-effectiveness of a music intervention targeting delirium in the neurosurgical setting. However, several limitations must be acknowledged. First, an important caveat to bear in mind is that our findings were not statistically significant, which may be due to low statistical power. The initial trial underlying this economic evaluation was relatively large compared to other trials done in this field. However, we partly based the current analyses on long-term data, including questionnaires, which led to loss to follow-up. This was expected, as these patients are vulnerable, cope with post-surgical treatments, and may have a poor prognosis. As a result, fewer data were available for the cost-effectiveness analysis over the entire follow-up period. Hence, our results should be interpreted as exploratory and hypothesis-generating only. Second, after randomization, the proportion of patients with a psychiatric history was lower in the music group. This imbalance could have led to an overestimation of the effect of the music intervention, since a lower incidence of delirium may be expected in a group with fewer psychiatric comorbidities. However, this was coped with by excluding patients with a psychiatric history, showing a similar trend between the two intervention groups. Third, the generalisability of the results may have been limited by the single-centre design of the study. We recommend future studies to analyse the effectiveness and cost-effectiveness of music interventions in multicentre randomized controlled trials with larger sample sizes.

## Conclusions

Perioperative music shows potential as a cost-effective intervention in the neurosurgical setting, as healthcare utilization during the primary admission seemed to be lower in patients receiving perioperative music, leading to reduced costs. This economic benefit may be linked to the decreased incidence of delirium observed in the music group. Cost-saving effects were not found in the longer-term follow-up data until 6 months after discharge. Further research is needed to build on the preliminary evidence from this study and confirm the promise of music as a cost-effective intervention for neurosurgical patients.

## Supplementary information

Below is the link to the electronic supplementary material.

Supplementary Material 1 (56.4 KB)

## Data Availability

Research data is available upon reasonable request.

## Abbreviations

CEA: Cost-Effectiveness Analysis
CHEERS: Consolidated Health Economic Evaluation Reporting Standards
DOSS: Delirium Observational Screening Scale
DSM-V: Diagnostic and Statistical Manual of Mental Disorders, Fifth Edition
HELP: Hospital Elder Life Program
ICER: Incremental Cost Effectiveness Ratio
iMCQ: iMTA Medical Consumption Questionnaire
iPCQ: iMTA Productivity Cost Questionnaire
LOS: Length Of Stay
mITT: modified Intention-To-Treat population
MOCA: Montreal Cognitive Assessment tool
POSH: Perioperative Optimization of Senior Health program
