# Supplementary material for: The effect of music interventions compared to standard-of-care on the prevention of delirium in neurosurgical patients: an analysis of costs and cost-effectiveness based on the MUSYC-trial
Source: Acta Neurochir (Wien). 2025 Feb 14;167(1):46. doi: 10.1007/s00701-025-06448-0 (PMC11828790; doi:10.1007/s00701-025-06448-0)
Supplement: Supplementary file 1 — Supplementary Material 1 (56.4 KB) [file 701_2025_6448_MOESM1_ESM.docx]

**Supplementary table S1. Consolidated Health Economic Evaluation Reporting Standards (CHEERS)**

| **Topic** | **No.** | **Item** | **Location where item is reported** |
| --- | --- | --- | --- |
| **Title** |  |  |  |
|  | 1 | Identify the study as an economic evaluation and specify the interventions being compared. | 1 |
| **Abstract** |  |  |  |
|  | 2 | Provide a structured summary that highlights context, key methods, results, and alternative analyses. | 2 |
| **Introduction** |  |  |  |
| **Background and objectives** | 3 | Give the context for the study, the study question, and its practical relevance for decision making in policy or practice. | 3,4 |
| **Methods** |  |  |  |
| **Health economic analysis plan** | 4 | Indicate whether a health economic analysis plan was developed and where available. | Study protocol (*) |
| **Study population** | 5 | Describe characteristics of the study population (such as age range, demographics, socioeconomic, or clinical characteristics). | 5 & previous article about this trial (**) |
| **Setting and location** | 6 | Provide relevant contextual information that may influence findings. | 5,6,7 |
| **Comparators** | 7 | Describe the interventions or strategies being compared and why chosen. | 5 |
| **Perspective** | 8 | State the perspective(s) adopted by the study and why chosen. | 5 |
| **Time horizon** | 9 | State the time horizon for the study and why appropriate. | 5 |
| **Discount rate** | 10 | Report the discount rate(s) and reason chosen. | 5 |
| **Selection of outcomes** | 11 | Describe what outcomes were used as the measure(s) of benefit(s) and harm(s). | 5,6 |
| **Measurement of outcomes** | 12 | Describe how outcomes used to capture benefit(s) and harm(s) were measured. | 5,6 |
| **Valuation of outcomes** | 13 | Describe the population and methods used to measure and value outcomes. | 5,6,7 |
| **Measurement and valuation of resources and costs** | 14 | Describe how costs were valued. | 6 |
| **Currency, price date, and conversion** | 15 | Report the dates of the estimated resource quantities and unit costs, plus the currency and year of conversion. | Supplementary Table 2 |
| **Rationale and description of model** | 16 | If modelling is used, describe in detail and why used. Report if the model is publicly available and where it can be accessed. | Not applicable |
| **Analytics and assumptions** | 17 | Describe any methods for analysing or statistically transforming data, any extrapolation methods, and approaches for validating any model used. | 7 |
| **Characterising heterogeneity** | 18 | Describe any methods used for estimating how the results of the study vary for subgroups. | Not applicable |
| **Characterising distributional effects** | 19 | Describe how impacts are distributed across different individuals or adjustments made to reflect priority populations. | 6 |
| **Characterising uncertainty** | 20 | Describe methods to characterise any sources of uncertainty in the analysis. | 8 |
| **Approach to engagement with patients and others affected by the study** | 21 | Describe any approaches to engage patients or service recipients, the general public, communities, or stakeholders (such as clinicians or payers) in the design of the study. | Not applicable |
| **Results** |  |  |  |
| **Study parameters** | 22 | Report all analytic inputs (such as values, ranges, references) including uncertainty or distributional assumptions. | 8 |
| **Summary of main results** | 23 | Report the mean values for the main categories of costs and outcomes of interest and summarise them in the most appropriate overall measure. | 8 and table 3 |
| **Effect of uncertainty** | 24 | Describe how uncertainty about analytic judgments, inputs, or projections affect findings. Report the effect of choice of discount rate and time horizon, if applicable. | 8,9,10,11 |
| **Effect of engagement with patients and others affected by the study** | 25 | Report on any difference patient/service recipient, general public, community, or stakeholder involvement made to the approach or findings of the study | Not applicable |
| **Discussion** |  |  |  |
| **Study findings, limitations, generalisability, and current knowledge** | 26 | Report key findings, limitations, ethical or equity considerations not captured, and how these could affect patients, policy, or practice. | 9,10,11 |
| **Other relevant information** |  |  |  |
| **Source of funding** | 27 | Describe how the study was funded and any role of the funder in the identification, design, conduct, and reporting of the analysis | 11 |
| **Conflicts of interest** | 28 | Report authors conflicts of interest according to journal or International Committee of Medical Journal Editors requirements. | 11 |

From: Husereau D, Drummond M, Augustovski F, et al. Consolidated Health Economic Evaluation Reporting Standards 2022 (CHEERS 2022) Explanation and Elaboration: A Report of the ISPOR CHEERS II Good Practices Task Force. Value Health 2022;25.

* Kappen P, Jeekel J, Dirven CMF, Klimek M, Kushner SA, Osse RJ, et al. Music to prevent deliriUm during neuroSurgerY (MUSYC) Clinical trial: a study protocol for a randomised controlled trial. BMJ Open. 2021;11(10):e048270.

** Kappen P, Mos MI, Johannes J, Clemens MFD, Steven AK, Robert-Jan O, et al. Music to prevent deliriUm during neuroSurgerY (MUSYC): a single-centre, prospective randomised controlled trial. BMJ Open. 2023;13(6):e069957.

**Supplementary Table S2. Assessment of costs, source and unit**

| **Cost Category** | **Unit** | **Source of value** | **Year of valuation** | **Cost price 2021 (€) **** |
| --- | --- | --- | --- | --- |
| **Radiology** |  |  |  |  |
| MRI of the brain | unit | Nederlandse Zorgautoriteit (NZa)* | 2014 | 230.43 |
| MRI of the spine | unit | Nederlandse Zorgautoriteit (NZa)* | 2014 | 233.34 |
| CT of the brain | unit | Nederlandse Zorgautoriteit (NZa)* | 2014 | 144.52 |
| CT of thorax/abdomen | unit | Nederlandse Zorgautoriteit (NZa)* | 2014 | 379.74 |
| Chest X-ray | unit | Nederlandse Zorgautoriteit (NZa)* | 2014 | 49.09 |
| **Small laboratory examinations** |  |  |  |  |
| Leukocytes | unit | Nederlandse Zorgautoriteit (NZa)* | 2014 | 1.93 |
| Thrombocytes | unit | Nederlandse Zorgautoriteit (NZa)* | 2014 | 1.93 |
| Haemoglobin | unit | Nederlandse Zorgautoriteit (NZa)* | 2014 | 1.93 |
| eGFR | unit | Nederlandse Zorgautoriteit (NZa)* | 2014 | 7.31 |
| Creatinine | unit | Nederlandse Zorgautoriteit (NZa)* | 2014 | 1.98 |
| Sodium | unit | Nederlandse Zorgautoriteit (NZa)* | 2014 | 1.98 |
| Potassium | unit | Nederlandse Zorgautoriteit (NZa)* | 2014 | 1.98 |
| Urea | unit | Nederlandse Zorgautoriteit (NZa)* | 2014 | 1.80 |
| Total costs of small examination | unit | Aggregated using previous values | 2014 | 20.84 |
| **Extensive laboratory examinations** |  |  |  |  |
| Glucose | unit | Nederlandse Zorgautoriteit (NZa)* | 2014 | 1.98 |
| Sodium | unit | Nederlandse Zorgautoriteit (NZa)* | 2014 | 1.98 |
| Potassium | unit | Nederlandse Zorgautoriteit (NZa)* | 2014 | 1.98 |
| Chloride | unit | Nederlandse Zorgautoriteit (NZa)* | 2014 | 1.93 |
| Calcium | unit | Nederlandse Zorgautoriteit (NZa)* | 2014 | 2.16 |
| Arterial blood gas | unit | Nederlandse Zorgautoriteit (NZa)* | 2014 | 6.18 |
| Lactate | unit | Nederlandse Zorgautoriteit (NZa)* | 2014 | 2.16 |
| Haemoglobin | unit | Nederlandse Zorgautoriteit (NZa)* | 2014 | 1.93 |
| Haematocrit | unit | Nederlandse Zorgautoriteit (NZa)* | 2014 | 1.93 |
| Blood type assessment | unit | Nederlandse Zorgautoriteit (NZa)* | 2014 | 3.53 |
| Total costs of extensive examination | unit | Aggregated using previous values | 2014 | 25.76 |
| **Medication** |  |  |  | (€/mg) |
| Naproxen | per milligram | Zorginstituut Nederland. 2023 ¶ | 2023 | 0.06/250 |
| Oxynorm | per milligram | Zorginstituut Nederland. 2023 ¶ | 2023 | 0.23/5 |
| Oxycontin | per milligram | Zorginstituut Nederland. 2023 ¶ | 2023 | 0.24/5 |
| Haloperidol | per milligram | Zorginstituut Nederland. 2023 ¶ | 2023 | 0,13/1 |
| Alprazolam | per milligram | Zorginstituut Nederland. 2023 ¶ | 2023 | 0.05/0.25 |
| Diazepam | per milligram | Zorginstituut Nederland. 2023 ¶ | 2023 | 0.03/5 |
| Lormetazepam | per milligram | Zorginstituut Nederland. 2023 ¶ | 2023 | 0.21/1 |
| Oxazepam | per milligram | Zorginstituut Nederland. 2023 ¶ | 2023 | 0.03/10 |
| Midazolam | per milligram | Zorginstituut Nederland. 2023 ¶ | 2023 | 0.13/7.5 |
| Temazepam | per milligram | Zorginstituut Nederland. 2023 ¶ | 2023 | 0.08/20 |
| Zolpidem | per milligram | Zorginstituut Nederland. 2023 ¶ | 2023 | 0.05/10 |
| Zopiclone | per milligram | Zorginstituut Nederland. 2023 ¶ | 2023 | 0.04/7.5 |
| **Consultations (N) §** |  |  |  |  |
| Psychiatry | consultation | NFU CAO ‡ ; Hakkaart- van Roijen et al. 2016 † | 2022 | 97.20 |
| Internal Medicine | consultation | NFU CAO ‡ ; Hakkaart- van Roijen et al. 2016 † | 2022 | 64.80 |
| Rehabilitation | consultation | NFU CAO ‡ ; Hakkaart- van Roijen et al. 2016 † | 2022 | 64.80 |
| Other medical | consultation | NFU CAO ‡ ; Hakkaart- van Roijen et al. 2016 † | 2022 | 19.50 |
| Dietetics | consultation | NFU CAO ‡ ; Hakkaart- van Roijen et al. 2016 † | 2022 | 19.50 |
| Physiotherapy | consultation | NFU CAO ‡ ; Hakkaart- van Roijen et al. 2016 † | 2022 | 19.50 |
| Ergotherapy | consultation | NFU CAO ‡ ; Hakkaart- van Roijen et al. 2016 † | 2022 | 19.50 |
| Speech therapy | consultation | NFU CAO ‡ ; Hakkaart- van Roijen et al. 2016 † | 2022 | 19.50 |
| **Outpatient consultations** |  |  |  |  |
| General practitioner | consultation | CBS ^1^; ZIN ^2^ | 2015 | 36.86 |
| Occupational physician | consultation | Hakkaart- van Roijen et al. † ;Arbo Unie CAO ^3^ | 2022 | 56.16 |
| Social worker | consultation | Hakkaart- van Roijen et al. † | 2016 | 72.61 |
| Psychologist or Psychiatrist | consultation | Zorginstituut Nederland ^2^ | 2015 | 71.49 |
| **Homecare** |  |  |  |  |
| Household aid | per hour | Hakkaart- van Roijen et al. † ; Zorginstituut Nederland ^2^ | 2015 | 25.70 |
| Personal care | per hour | Hakkaart- van Roijen et al. † ; Zorginstituut Nederland ^2^ | 2015 | 55.85 |
| Nursing | per hour | Hakkaart- van Roijen et al. † ; Zorginstituut Nederland ^2^ | 2015 | 81.55 |
| Combination ^5^ | per hour | Hakkaart- van Roijen et al. † ; Zorginstituut Nederland ^2^ | 2015 | 54.37 |
| **Visit to institution** |  |  |  |  |
| Residential care facility | per visit | NZa ^4^ ; Zorginstituut Nederland ^2^ | 2015 | 74.85 |
| Rehabilitation centre | per visit | NZa ^4^ ; Zorginstituut Nederland ^2^ | 2015 | 170.92 |
| Psychiatric Unit | per visit | NZa ^4^ ; Zorginstituut Nederland ^2^ | 2015 | 109.48 |
| **Overnight Stay in institution** |  |  |  |  |
| Residential care facility | per overnight stay | Hakkaart- van Roijen et al. † ; NZa ^6^; ZIN^2^ | 2015 | 187.67 |
| Rehabilitation centre | per overnight stay | Hakkaart- van Roijen et al. † ; NZa ^6^; ZIN^2^ | 2015 | 513.86 |
| Psychiatric Unit | per overnight stay | Hakkaart- van Roijen et al. † ; NZa ^6^; ZIN^2^ | 2015 | 337.76 |
| **Time of informal caregivers** |  |  |  |  |
| Homecare | per hour | Hakkaart- van Roijen et al. † ; CAK^7^ | 2014 | 15.64 |
| Personal care | per hour | Hakkaart- van Roijen et al. † ; CAK^7^ | 2014 | 15.64 |
| Practical care | per hour | Hakkaart- van Roijen et al. † ; CAK^7^ | 2014 | 15.64 |
| Combination care | per hour | Hakkaart- van Roijen et al. † ; CAK^7^ | 2014 | 15.64 |
| **Productivity** |  |  |  |  |
| Absenteeism | per hour | Hakkaart- van Roijen et al. † ; Krol et al. ^8^ ; CBS ^9^ | 2014 | 38.97 |
| Presenteeism | per hour | Hakkaart- van Roijen et al. † ; Krol et al. ^8^ ; CBS ^9^ | 2014 | 38.97 |
| Unpaid work | per hour | Hakkaart- van Roijen et al. † ; CAK ^7^ | 2014 | 15.64 |

*NZa. (2014b). Tarievenlijst eerstelijnsdiagnostiek, TB/CU-7078-01. ().NZa. <http://www.nza.nl/regelgeving/bijlagen/Bijlage_1_bij_TB_CU_7078_01_Tarievenlijst_Eerstelijnsdiagnostiek>

¶ Zorginstituut Nederland. 2023. https://www.medicijnkosten.nl. Retrieved 31-05-2023, from <https://www.medicijnkosten.nl>

‡ NFU. (2022). Cao Universitair Medische Centra 2022-2023; Utrecht. <https://www.nfu.nl/sites/default/files/2022-04/cao_umc-NL2022-2023_April.pdf>

† Hakkaart- van Roijen, L., van der Linden, N., Bouwmans, C., Kanters, T., & Swan Tan, S. (2016). Kostenhandleiding: Methodologie voor kostenonderzoek methoden en referentieprijzen voor economische evaluaties in de gezondheidszorg. ( No. 90). Diemen: Zorginstituut Nederland. 10.1007/s12508-012-0128-3. <https://www.narcis.nl/publication/RecordID/oai:pure.eur.nl:publications%2Fb9e216a0-92f0-4cb1-b894-9bbf94e46e10>

§ A distinction is made between medical specialists (i.e., surgeons, anesthesiologist, psychiatrist, internist, and rehabilitation doctors) and other health care staff (i.e., dieticians, physical therapists, ergotherapists and speech therapists).

^1^ CBS. (2014a). Bevolking. https://www.zorgcijfersdatabank.nl/. Retrieved 31-05-2023, from https://www.zorgcijfersdatabank.nl/ ; CBS. (2014b). Medische contacten. https://www.zorgcijfersdatabank.nl/. Retrieved 31-05-2023, from <https://www.zorgcijfersdatabank.nl/>

^2^ ZIN. (2015). Zorgcijfersdatabank. 31-05-2023. <https://www.zorgcijfersdatabank.nl/>

^3^ Arbo Unie. (2022). Collectieve arbeidsovereenkomst Arbo Unie. Utrecht: Arbo Unie. <https://www.lad.nl/wp-content/uploads/2022/06/Cao-Arbo-Unie-2022_14-06.pdf>

^4^ NZa. (2014a). Prestatiebeschrijvingen en tarieven dagbesteding en vervoer AWBZ, CA-300-582. Utrecht: NZa. [http://www.nza.nl/1048076/1048090/CA_300_582__Prestatiebeschrijvingen_en_tarieven_dagbesteding_en_ vervoer_AWBZ.pdf](http://www.nza.nl/1048076/1048090/CA_300_582__Prestatiebeschrijvingen_en_tarieven_dagbesteding_en_%20vervoer_AWBZ.pdf)

^5^ The follow-up was gathered with two electronic data-systems: Limesurvey and Openclinica. In Openclinica four patients answered having a combination of occupational care, without entering of which three options the combination existed. To include these patients, a combination price was calculated. Explanation: *=E(x)= ¼ (1/2 * price of household assistance + ½ * price of personal care + ½ * price of nursing care + 1/3 + sum of all three) = 54.37

^6^ NZa. (2015). Tariefbeschikking generalistische basis GGZ, TB/CU-5069. (). Utrecht: NZa.

<http://www.nza.nl/regelgeving/tarieven/?selectedCategory=124431&selectedTextItem=133321>

^7^ CAK. (2014). Uurtarieven. Retrieved 31-05-2023, from

[https://www.hetcak.nl/portalserver/portals/cak-portal/pages/k1-2-9-4-uurtarie- ven.html](https://www.hetcak.nl/portalserver/portals/cak-portal/pages/k1-2-9-4-uurtarie-%20ven.html)

^8^ Krol, M., & Brouwer, W. (2014). How to estimate productivity costs in economic evaluations. PharmacoEconomics, 32(4), 335-344. 10.1007/s40273-014-0132-3

^9^ CBS. (2023a). Prijsindex arbeid. Retrieved 31-05-2023, from <https://www.cbs.nl/nl-nl/onze-diensten/methoden/onderzoeksomschrijvingen/korteonderzoeksbeschrijvingen/prijsindex-arbeid>

**Supplementary Table S3. Costs of the music intervention**

| **Type** | **N** | **Costs per device** | **Total costs** | **Total costs per 5 years** |
| --- | --- | --- | --- | --- |
| Sony WH-CH700N Headphones | 5 | 101.64 | 508.20 | 508.20 |
| JBL T110BT Earphones | 5 | 35.99 | 179.95 | 179.95 |
| HAMA In-ear Earbuds | 34 | 6.66 per 12 items | 226.44 | 226.44 |
| HAMA 48910 Audio extension cable | 5 | 7.98 | 39.90 | 39.90 |
| Alcatel 1T7 Family 16 GB Tablet | 5 | 75.00 | 375.00 | 375.00 |
| Private Copying Levy |  |  | 15.75 | 15.75 |
| Music streaming application | 1 |  | 14.99 per month | 899.40 |
| All Items |  |  |  | 2244.64 |
|  |  |  |  |  |
|  |  |  |  |  |
| Study period |  |  |  | 1.43 years |
| Number of patients in music group |  |  |  | 91 |
| Extrapolated number of patients in music group in 5 years |  |  |  | 319 |
| Cost per patient for music intervention |  |  |  | 7.04** |

All costs are reported in euros (€). The products used for the intervention were estimated to have a lifespan of five years. The headphones, earphones, audio extension cables, and tablets were reused between patients after being thoroughly cleaned following each use. The in-ear earbuds of the earphones used intraoperatively were disposed of after each patient’s use. Personnel costs for the intervention were assumed to be minor since staff members did not need to be present while patients were undergoing the music intervention. Therefore, these costs were not included.

**Supplementary Table S4. Extension of healthcare utilization data**

|  | **Control** | | |  | **Music** | | **P-value** |
| --- | --- | --- | --- | --- | --- | --- | --- |
|  | N* | Total number (Mean) | Median (IQR) | N* | Total number (Mean) | Median (IQR) |  |
| **Direct medical care** |  |  |  |  |  |  |  |
| Operation (minutes): |  |  |  |  |  |  |  |
| Time in operation room | 93 | 30571 (328.72) | 283 (226.50-405) | 91 | 28046 (308.20) | 281 (205-358) | 0.299 |
| Incision duration | 93 | 23925 (257.26) | 218 (159-329) | 91 | 21280 (233.85) | 213 (140-288) | 0.176 |
| Hospital length of stay (days): |  |  |  |  |  |  |  |
| Nursing Department | 93 | 565 (6.08) | 3 (2-6) | 91 | 460 (5.05) | 3 (2-6) | 0.792 |
| Post-anaesthesia care unit | 93 | 101 (1.09) | 1 (1-1) | 91 | 100 (1.10) | 1 (1-1) | 0.385 |
| Intensive Care Unit | 93 | 22 (0.24) | 0 (0-0) | 91 | 21 (0.23) | 0 (0-0) | 0.557 |
| Radiology: |  |  |  |  |  |  |  |
| MRI | 93 | 95 (1.02) | 1 (1-1) | 91 | 92 (1.01) | 1 (1-1) | 0.982 |
| CT | 93 | 107 (1.15) | 1 (0-1) | 91 | 91 (1.0) | 1 (0-1) | 0.279 |
| X-rays | 93 | 16 (0.17) | 0 (0-0) | 91 | 11 (0.12) | 0 (0-0) | 0.535 |
| Laboratory test †: |  |  |  |  |  |  |  |
| Basic Examinations | 93 | 230 (2.47) | 1 (1-2) | 91 | 183 (2.01) | 1 (1-2) | 0.42 |
| Extended Examinations | 93 | 668 (7.18) | 5 (4-7) | 91 | 761 (8.36) | 5 (4-7) | 0.876 |
| Medical consultations: |  |  |  |  |  |  |  |
| Psychiatry | 93 | 62 (0.67) | 0 (0-0) | 91 | 32 (0.35) | 0 (0-0) | 0.38 |
| Internal Medicine | 93 | 19 (0.2) | 0 (0-0) | 91 | 21 (0.23) | 0 (0-0) | 0.308 |
| Rehabilitation | 93 | 31 (0.33) | 0 (0-0) | 91 | 18 (0.2) | 0 (0-0) | 0.288 |
| Other § | 93 | 42 (0.45) | 0 (0-0) | 91 | 26 (0.29) | 0 (0-0) | 0.164 |
| Total medical consultations | 93 | 154 (1.66) | 0 (0-1) | 91 | 97 (1.07) | 0 (0-0) | 0.548 |
| Paramedical consultations: |  |  |  |  |  |  |  |
| Dietetics | 93 | 21 (0.23) | 0 (0-0) | 91 | 22 (0.24) | 0 (0-0) | 0.278 |
| Physiotherapy | 93 | 192 (2.06) | 0 (0-2) | 91 | 139 (1.53) | 0 (0-2) | 0.639 |
| Ergotherapy | 93 | 4 (0.04) | 0 (0-0) | 91 | 4 (0.04) | 0 (0-0) | 0.173 |
| Speech therapy | 93 | 25 (0.27) | 0 (0-0) | 91 | 14 (0.15) | 0 (0-0) | 0.789 |
| Total paramedical consultations | 93 | 242 (2.6) | 1 (0-2) | 91 | 179 (1.97) | 0 (0-0) | 0.62 |
| Medication (milligrams) ¶: |  |  |  |  |  |  |  |
| Naproxen | 93 | 10250 (110.22) | 0 (0-0) | 91 | 5750 (63.19) | 0 (0-0) | 0.059 |
| Oxynorm | 93 | 485 (5.22) | 0 (0-5) | 91 | 315 (3.46) | 0 (0-5) | 0.984 |
| Oxycontin | 93 | 280 (3.01) | 0 (0-0) | 91 | 350 (3.85) | 0 (0-0) | 0.689 |
| Haloperidol | 93 | 149.5 (1.61) | 0 (0-0) | 91 | 34 (0.37) | 0 (0-0) | 0.519 |
| Benzodiazepines | 93 | 478 (5.14) | 0 (0-0) | 91 | 111 (1.22) | 0 (0-0) | 0.084 |
| **Indirect medical care** |  |  |  |  |  |  |  |
| Readmissions (number) ‡ | 93 | 21 (0.23) | 0 (0-0) | 91 | 34 (0.37) | 0 (0-0) | 0.832 |
| Readmission days ‡ | 93 | 83 (0.89) | 0 (0-0) | 91 | 169 (1.86) | 0 (0-0) | 0.796 |
| Reoperations ‡ | 93 | 14 (0.15) | 0 (0-0) | 91 | 16 (0.18) | 0 (0-0) | 0.767 |
| Radiology |  |  |  |  |  |  |  |
| MRI | 93 | 93 (1) | 1 (0.5-1.0) | 91 | 98 (1.08) | 1 (0-2) | 0.532 |
| CT | 93 | 32 (0.34) | 0 (0-0) | 91 | 40 (0.44) | 0 (0-1) | 0.654 |
| X-rays | 93 | 1 (0.1) | 0 (0-0) | 91 | 0 (0) | 0 (0-0) | 0.323 |
| Outpatient consultations: |  |  |  |  |  |  |  |
| General practitioner | 38 | 134 (3.53) | 3 (1-5.25) | 37 | 209 (5.65) | 3 (1-5.50) | 0.894 |
| Occupational physician | 38 | 47 (1.24) | 0 (0-3) | 37 | 46 (1.24) | 0 (0-3) | 0.822 |
| Social worker | 38 | 20 (0.53) | 0 (0-0) | 37 | 27 (0.73) | 0 (0-0) | 0.927 |
| Psychotherapist, Psychiatrist | 38 | 84 (2.21) | 0 (0-0) | 37 | 45 (1.22) | 0 (0-0) | 0.841 |
| Homecare (hours): |  |  |  |  |  |  |  |
| Household aid | 38 | 39 (1.03) | 0 (0-0) | 37 | 252 (6.81) | 0 (0-0) | 0.083 |
| Personal care | 38 | 82 (2.16) | 0 (0-0) | 37 | 174 (4.7) | 0 (0-0) | 0.543 |
| Nursing | 38 | 12 (0.32) | 0 (0-0) | 37 | 4 (0.1) | 0 (0-0) | 0.574 |
| Combination | 38 | 228 (6.0) | 0 (0-0) | 37 | 0 (0) | 0 (0-0) | 0.324 |
| Visit to institutions (days): |  |  |  |  |  |  |  |
| Residential care facility | 38 | 0 (0) | 0 (0-0) | 37 | 0 (0) | 0 (0-0) | 1 |
| Rehabilitation centre | 38 | 48 (1.26) | 0 (0-0) | 37 | 101 (2.73) | 0 (0-0) | 0.096 |
| Psychiatric Unit | 38 | 1 (0.26) | 0 (0-0) | 37 | 6 (0.16) | 0 (0-0) | 0.97 |
| Other | 38 | 4 (0.11) | 0 (0-0) | 37 | 40 (1.08) | 0 (0-0) | 0.522 |
| Overnight Stay in institution (days): |  |  |  |  |  |  |  |
| Residential care facility | 38 | 51 (1.34) | 0 (0-0) | 37 | 0 (0) | 0 (0-0) | 0.16 |
| Rehabilitation centre | 38 | 40 (1.05) | 0 (0-0) | 37 | 2 (0.05) | 0 (0-0) | 1 |
| Psychiatric Unit | 38 | 0 (0) | 0 (0-0) | 37 | 0 (0) | 0 (0-0) | 1 |
| Other | 38 | 0 (0) | 0 (0-0) | 37 | 0 (0) | 0 (0-0) | 1 |
| **Direct non-medical care** |  |  |  |  |  |  |  |
| Time of informal caregivers (hours): |  |  |  |  |  |  |  |
| Homecare | 38 | 2109 (55.5) | 0 (0-27.75) | 37 | 1728 (46.70) | 0 (0-49.5) | 0.473 |
| Personal care | 38 | 299 (7.87) | 0 (0-0) | 37 | 167 (4.51) | 0 (0-0) | 0.737 |
| Practical care | 38 | 895 (23.55) | 0 (0-3.75) | 37 | 1356 (36.65) | 0 (0-21) | 0.357 |
| Combination care | 38 | 260 (6.84) | 0 (0-0) | 37 | 3379 (91.32) | 0 (0-0) | 0.248 |

P-values were calculated using Mann-Whitney U tests, due to non-parametric data. Bonferroni-corrected alpha is 0.0001, e.g. p-values smaller than 0.0001 is defined statistically significant. *number of patients with available data per variable † To streamline data extraction and valuation of laboratory tests the examinations have been categorized into small laboratory examinations (< 10 values) and large laboratory examinations (> 10 values) § including other medical consultations, which are related to neurosurgical indication and/or (consequences of) delirium ¶ Dose of administered medication in milligrams ‡ Data of Erasmus Medical Centre, data of readmissions or reoperations in other centres are unknown
